# 免疫亲和柱净化-超高效液相色谱-三重四极杆质谱法高灵敏测定尿液和血浆中3种鹅膏毒肽

**DOI:** 10.3724/SP.J.1123.2021.08018

**Published:** 2022-05-08

**Authors:** Xiuyao ZHANG, Xinxin CAI, Xiaoyi ZHANG, Ruifen LI, Yunfeng ZHAO

**Affiliations:** 1.温州市疾病预防控制中心, 浙江 温州 325001; 1. Wenzhou Municipal Center for Disease Control and Prevention, Wenzhou 325001, China; 2.国家食品安全风险评估中心, 北京 100022; 2. China National Center for Food Safety Risk Assessment, Beijing 100022, China

**Keywords:** 超高效液相色谱-三重四极杆质谱, 免疫亲和柱净化, 鹅膏毒肽, 尿液, 血浆, ultra performance liquid chromatography-triple quadrupole mass spectrometry (UPLC-MS/MS), quadrupole-time-of-flight high resolution mass spectrometry (Q-TOF HRMS), amanitins, urine, plasma

## Abstract

建立了超高效液相色谱-三重四极杆质谱高灵敏测定尿液和血浆中*α*-鹅膏毒肽、*β*-鹅膏毒肽和*γ*-鹅膏毒肽的方法。经过免疫亲和柱净化,尿液样品浓缩20倍、血浆样品浓缩10倍,以Kinetex Biphenyl色谱柱(100 mm×2.1 mm, 1.7 μm)作为分析柱,甲醇-0.005%(v/v)甲酸水溶液作为流动相进行梯度洗脱分离,电喷雾电离、负离子、多反应监测模式下检测,外标法定量。3种鹅膏毒肽的线性范围为0.1~200 ng/mL,相关系数(*r*)>0.999。尿液和血浆中3种鹅膏毒肽的基质效应和提取回收率分别为92%~108%和90%~103%,变异系数均小于13%。尿液中3种鹅膏毒肽的准确度为-9.4%~8.0%,重复性和中间精度分别为3.0%~14%和3.5%~18%,当取样量为2.00 mL时,方法的检出限均为0.002 ng/mL;血浆中3种鹅膏毒肽的准确度为-13%~8.0%,重复性和中间精度分别为3.9%~9.7%和5.5%~12%,当取样量为1.00 mL时,方法的检出限均为0.004 ng/mL。该法操作简单、灵敏、准确,已在中毒患者摄入野生蘑菇后138 h的尿液中检出0.0067 ng/mL *α*-鹅膏毒肽和0.0059 ng/mL *β*-鹅膏毒肽。该法已成功解决中毒患者尿液和血浆中超痕量鹅膏毒肽的检测难题,对于疑似中毒病人的早诊断、早治疗、降低死亡率都具有非常重要意义,也为今后开展此类毒素毒理作用及机体代谢规律的研究提供了可靠的技术支撑。

## Abstract (en)

Cases of toxic mushroom poisoning occur frequently in China every year. In particular, mushrooms containing amanitins can cause acute liver damage, with high mortality rates. The symptoms of acute liver damage are experienced 9-72 h after consumption of the mushrooms. At this time, the concentration of amanitins in blood and urine is too low to be detected even by the highly sensitive ultra performance liquid chromatography-triple quadrupole mass spectrometry (UPLC-MS/MS), thus rendering clinical diagnosis and treatment difficult. To this end, a method was developed for the determination of *α*-amanitin, *β*-amanitin and *γ*-amanitin in urine and plasma by UPLC-MS/MS. Urine and plasma samples were extracted and cleaned up by using an immunoaffinity column. A sample of 2.00 mL urine or 1.00 mL of plasma was diluted with 8.00 mL of phosphate buffer solution (PBS) and then loaded onto the immunoaffinity column at a flow rate of 0.5-1.0 mL/min. After washing the column with 10 mL of PBS and 13 mL of water successively, the bound amanitins were eluted with 3.00 mL of methanol-acetone (1∶1, v/v). The eluent was dried under nitrogen at 55 ℃. The residue was dissolved in 100 μL of 10% (v/v) methanol aqueous solution. The amanitins in urine were concentrated 20 times, while those in plasma were concentrated 10 times. Chromatographic separation was performed on a Kinetex Biphenyl column (100 mm × 2.1 mm, 1.7 μm) with gradient elution using methanol and 0.005% (v/v) formic acid aqueous solution as mobile phases. The three amanitins were detected by negative electrospray ionization tandem mass spectrometry in the multiple reaction monitoring (MRM) mode and quantified by the solvent standard curve external standard method. Method validation was performed as recommended by the European Drug Administration (EMEA). Four levels of quality control (QC) samples were prepared, which covered the calibration curve range, viz., the limit of quantification (LOQ), within three times the LOQ (low QC), medium QC, and at 85% of the upper calibration curve range (high QC), and used to test the accuracy, precision, matrix effect, extraction recovery, and stability. The calibration curves for the three amanitins showed good linear relationships in the range of 0.1-200 ng/mL, and the correlation coefficients (*r*) were greater than 0.999. The matrix effects and extraction efficiencies of the three amanitins in urine and plasma were 92%-108% and 90%-103%, respectively, and the coefficients of variation were less than 13%. The accuracies of the three amanitins in urine were within -9.4%-8.0%. The repeatability and intermediate accuracies were 3.0%-14% and 3.5%-18%, respectively. When the sampling volume was 2.00 mL, the limits of detection of the three amanitins in urine were 0.002 ng/mL. The accuracies of the three amanitins in plasma were within -13%-8.0%. The repeatability and intermediate accuracies were 3.9%-9.7% and 5.5%-12%, respectively. When the sampling volume was 1.00 mL, the limits of detection of the three amanitins in plasma were 0.004 ng/mL. The developed method is simple, sensitive, and accurate. During toxic mushroom poisoning detection, 0.0067 ng/mL of *α*-amanitin and 0.0059 ng/mL of *β*-amanitin were detected in the urine of poisoned patients 138 h after ingesting poisonous mushrooms. This method has successfully solved the problem of detecting ultra-trace levels of amanitins in the urine and plasma of poisoned patients. It has important practical significance for the early diagnosis, early treatment, and mortality reduction of suspected poisoning patients. This method can also provide reliable technical support for future research on the toxicological effects and in vivo metabolism of these toxins.

毒蘑菇又称毒蕈,我国有480多种有毒蘑菇^[1]^,每年都有毒蘑菇中毒事件发生,以春夏季最为多见,常致人死亡。据报道^[2]^, 2020年我国24个省份发生了676起蘑菇中毒事件,共计1719人中毒,涉及约102种毒蘑菇,造成25人死亡,病死率为1.45%,其中17例由造成急性肝损害型的含鹅膏毒肽类(amatoxins)的毒蘑菇引起。引起中毒的蘑菇毒素种类较多,其中鹅膏毒肽类为致死的主要毒素,分布在鹅膏属(*Amanita*)、环柄菇属(*Lepiota*)和盔孢伞属(*Galerina*)等蘑菇中的一类双环八肽。目前,研究人员已经分离出9种鹅膏毒肽,其中*α*-、*β*-、*γ*-鹅膏毒肽(*α*-、*β*-、*γ*-amanitin)在毒蘑菇中含量最高、毒性最大,是引起肝损害型中毒的主要毒素。鹅膏毒肽类人类致死量约为0.1 mg/(kg·bw),它们能强烈抑制细胞RNA聚合酶的活性,从而阻碍蛋白质的合成,引起多种脏器细胞特别是肝细胞的坏死,导致中毒者6~16 d内死亡^[3⇓-5]^。

鹅膏毒肽引起的中毒往往要经历潜伏期(8~12 h)、胃肠炎期(8~48 h)、假愈期(48~72 h)和内脏损害期(72~96 h),通常在摄入9~72 h后才出现中毒症状^[3⇓⇓⇓-7]^,对于没有经验的医疗机构往往在病人摄入毒物数天后才能确诊。此时鹅膏毒肽已进入肝、肾等组织,血液和尿液中鹅膏毒肽含量很低,即便是高灵敏的液相色谱-三重四极杆质谱法也无法检出,我们曾用超高效液相色谱-三重四极杆质谱法检测了多起毒蘑菇中毒事件中数十份血液和尿液样品^[8]^,但均未检出鹅膏毒肽,而在引起中毒的野生蘑菇和含野生蘑菇的食物样本中检出了高浓度的鹅膏毒肽和鬼笔毒肽^[9,10]^。Jaeger等^[7]^从中毒初期患者的血浆和尿液中检出*α*-鹅膏毒肽和*β*-鹅膏毒肽,同时发现血浆和尿液中鹅膏毒肽的含量高度依赖于摄入毒物到采样的时间间隔,摄入鹅膏毒肽后,可分别在24 h和72 h内的血浆和尿液样本中检出鹅膏毒肽,超过这个时间间隔,样品中鹅膏毒肽含量往往较低,难以检出。文献^[11,12]^认为检测不出鹅膏毒肽的原因是检测方法的灵敏度不够。因此,开发建立一种检测尿液和血浆中鹅膏毒肽的超高灵敏度的确证检测方法,可以在中毒发生后更长的时间内检出鹅膏毒肽,对于疑似中毒病人的诊断、治疗、降低死亡率都具有非常重要的现实意义。

目前尿液和血浆中鹅膏毒肽类的检测方法主要有液相色谱-三重四极杆质谱法^[10,13⇓⇓⇓⇓⇓-19]^和液相色谱-高分辨质谱法^[20,21]^等,尿液的检出限为0.15~5 ng/mL,血浆的检出限为0.1~10 ng/mL。最近,徐小民等^[22]^报道了在线固相萃取-液相色谱-质谱联用检测尿液中*α*-鹅膏毒肽的方法,方法检出限达0.03 ng/mL,可以在中毒患者食用毒蘑菇72 h,甚至92 h后的尿液中检出*α*-鹅膏毒肽,并在9名急性肝损害型中毒患者尿液中检出*α*-鹅膏毒肽,含量分别为0.11~53.1 ng/mL。

本研究基于对超高效液相色谱分离条件、质谱测定条件的优化,提高了方法的灵敏度,特别是对免疫亲和柱净化条件的优化,达到了尿液和血浆中痕量毒素高灵敏检测的目的,为鹅膏毒肽类中毒的鉴别诊断以及确诊提供了可靠的技术支持。

## 1 实验部分

### 1.1 仪器与试剂

Acquity I-Class超高效液相色谱仪(美国Waters公司); QTRAP 6500三重四极杆/线性离子阱复合质谱仪(美国AB Sciex公司); MS3旋涡混旋器(德国IKA公司); Orion 5-star酸度计(美国Orion公司); 24孔N-EVAP氮吹仪(美国Organomation公司); Gradient A10 Mill-Q超纯水器(法国Millipore公司)。

甲醇和乙腈为色谱级(德国Merck公司);丙酮和甲酸为色谱级(美国Tedia公司);鹅膏毒肽免疫亲和柱(3 mL,北京华安麦科生物技术有限公司)。*α*-鹅膏毒肽、*β*-鹅膏毒肽、*γ*-鹅膏毒肽、羧基二羟鬼笔毒肽和二羟鬼笔毒肽标准品(纯度均大于90%,加拿大Alexis Biochemicals公司),用甲醇配制成100 μg/mL标准储备溶液,保存于-35 ℃冰箱中。磷酸盐缓冲溶液(PBS缓冲液,pH 7.3):称取12.90 g十二水合磷酸氢二钠、2.18 g二水合磷酸二氢钠、8.50 g氯化钠,用800 mL水溶解,调节pH至7.3,用水定容至1000 mL。健康人血浆经浙江省卫生行政部门审批后由温州市中心血站提供,尿液由健康志愿者提供。

### 1.2 标准溶液和质控样的制备

吸取各100 μL 3种鹅膏毒肽标准储备溶液(100 μg/mL),于10 mL容量瓶中,用10%(v/v)甲醇水溶液定容,先制成3种鹅膏毒肽混合标准使用溶液(1.00 μg/mL)。然后再用10%(v/v)甲醇水溶液稀释成3种鹅膏毒肽系列标准溶液(0.1、0.3、1.0、5.0、50和200 ng/mL)。

质控样品按欧洲药品管理局(EMEA)等要求制备^[23,24]^。每次测定时在空白尿液和血浆中分别加入3种鹅膏毒肽,制成定量限(LOQ)、低(3×LOQ)、中(medium QC)和高(为85%的测定上限,high QC)4个水平的质控样品,其中尿液质控样品的水平分别为0.005、0.015、0.25和8.5 ng/mL,血浆质控样品水平分别为0.01、0.030、0.50和17.0 ng/mL。用于进行准确度、精密度、基质效应、提取回收率和稳定性等试验。

### 1.3 样品前处理

尿样采集后保存于具塞塑料离心管中,于-20 ℃保存。全血样品采集于含抗凝剂的采血管中,以3000 r/min离心10 min,取上层血浆于塑料离心管中,于-20 ℃保存。

吸取2.00 mL尿样或1.00 mL血浆,于15 mL塑料具塞离心管中,加入8.00 mL PBS缓冲溶液,混匀,以0.5~1.0 mL/min的流速上样至免疫亲和柱,再分别用10 mL PBS缓冲溶液和13 mL水淋洗,抽干,用3.00 mL甲醇-丙酮(1∶1, v/v)溶液洗脱,洗脱液于55 ℃水浴中氮气吹干,加入100 μL 10%(v/v)甲醇水溶液溶解残渣,溶解液过0.22 μm滤膜,滤液转移至微量自动进样瓶中,待测。

### 1.4 色谱条件

分析柱为Kinetex Biphenyl色谱柱(100 mm×2.1 mm, 1.7 μm),保护柱为Security Guard ULTRA Kinetex Biphenyl(2.1 mm, 1.7 μm)(美国Phenomenex公司);柱温45 ℃;流动相A:甲醇,流动相B: 0.005%(v/v)甲酸水溶液;流速:0.300 mL/min。梯度洗脱程序:0~6.0 min, 5%A~50%A; 6.0~6.1 min, 50%A~95%A; 6.1~8.0 min, 95%A; 8.0~8.1 min, 95%A~5%A; 8.1~10.0 min, 5%A。进样体积:10 μL。

运行开始时,色谱柱流出液经六通切换阀流至废液;4.50~6.30 min,六通切换阀将柱流出液切换到质谱。

### 1.5 质谱条件

电喷雾电离源、负离子、多反应监测(MRM)模式检测。离子化电压(IS): -4500 V,离子源温度(TEM): 500 ℃,气帘气(CUR)压力:208 kPa,喷雾气(GS1)压力:312 kPa,辅助加热气压力(GS2): 483 kPa,碰撞气(CAD): High。其他参数见表1。

**表1:** 3种鹅膏毒肽的质谱参数

Compound	Precursor ion (*m/z*)	Product ion (*m/z*)	Declustering potential/ V	Collision energy/ eV	Retention time/ min
*α*-Amanitin	917.4	899.3^*^	-140	-36	5.04
		205.1		-64	
*β*-Amanitin	918.4	900.3^*^	-140	-38	4.88
		205.1		-66	
*γ*-Amanitin	901.4	883.3^*^	-140	-36	5.82
		205.1		-64	

* Quantitative ion.

## 2 结果与讨论

### 2.1 质谱条件的优化

文献报道的鹅膏毒肽测定条件多在电喷雾电离正离子模式下进行^[10,13⇓⇓⇓⇓⇓⇓-20]^,也少量报道在电喷雾电离负离子模式下进行^[22]^。利用针泵泵入鹅膏毒肽的标准溶液,在正负大气压化学电离方式下,3种鹅膏毒肽的响应值都较低。在电喷雾电离正离子模式下的质谱可见[M+H]^+^、[M+Na]^+^和[M+K]^+^峰,但它们的碎片离子不稳定,响应值也不高;而在电喷雾电离负离子模式下,出现了较强的[M-H]^-^峰,以[M-H]^-^作为母离子进行碰撞解离,通过子离子扫描得到目标化合物碎片离子的信息,见图1,响应值优于电喷雾电离正离子模式下的响应值。因此后续测定在电喷雾负离子方式下进行。

**图1:**
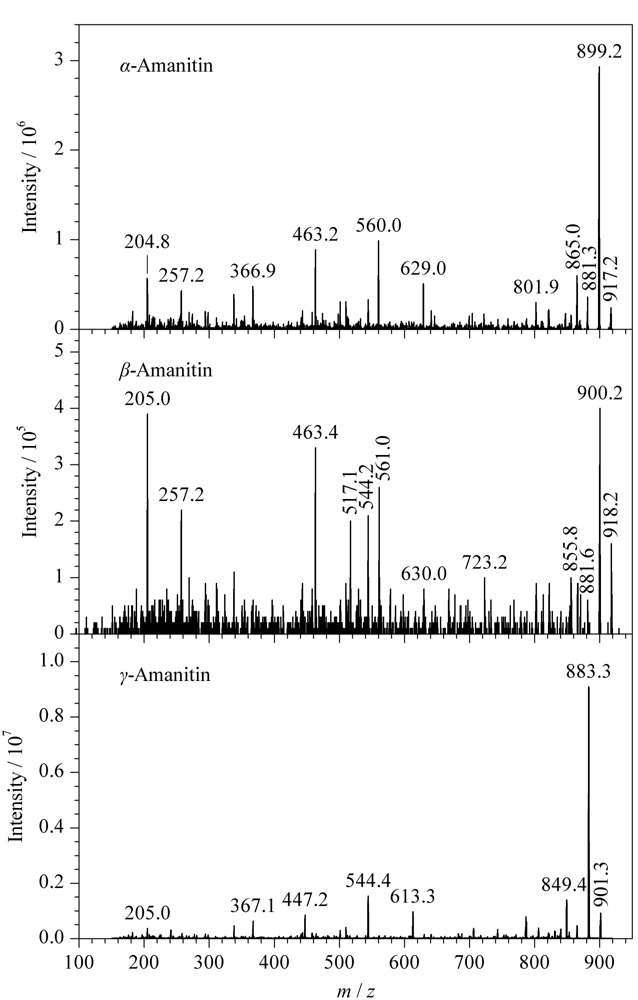
3种鹅膏毒肽的子离子扫描质谱图

实验对去簇电压、碰撞能量等参数进行优化,使得分子离子对的信号达到最高。每种待测物选择响应最高的两对分子离子对,以其中响应高的分子离子对作为定量离子对,另一分子离子对作为定性离子对。优化后的质谱条件见1.5节。

### 2.2 色谱条件的优化

鹅膏毒肽类的分离大多在近中性条件下进行^[10,13,15,18⇓-20]^,也有在酸性^[14]^或碱性^[25]^条件下分离,大部分选用反相液相色谱柱^[10,14⇓⇓⇓⇓⇓-20,25]^,也有选用亲水液相色谱柱^[13]^。本文首先采用通用性强的ACQUITY UPLC BEH C_18_色谱柱(100 mm×2.1 mm, 1.7 μm, Waters公司)进行分离优化,有机相分别采用甲醇和乙腈,水相分别采用水、2 mmol/L乙酸铵水溶液、0.05%氨水溶液和0.01%甲酸水溶液。实验发现,3种鹅膏毒肽的质谱响应对流动相的组成非常敏感,与甲醇-水或乙腈-水相比较,乙酸铵对3种鹅膏毒肽的质谱信号有明显的抑制作用,而0.05%氨水或0.01%甲酸水溶液对3种鹅膏毒肽的质谱信号有明显的增强作用。尝试了不同体积分数的氨水(0.01%~0.05%)作为水相,但是无论乙腈还是甲醇作为有机相时,3种鹅膏毒肽的质谱响应均非常不稳定。采用甲醇-0.01%甲酸水溶液或乙腈-0.01%甲酸水溶液作为流动相时,3种鹅膏毒肽的质谱信号稳定,其中以甲醇-0.01%甲酸水溶液响应值更高,但是*α*-鹅膏毒肽和*β*-鹅膏毒肽在BEH C_18_色谱柱上不能完全分离,而这2种鹅膏毒肽的母离子仅相差1 Da, *α*-鹅膏毒肽的M+1同位素峰在*β*-鹅膏毒肽的质谱通道中有明显的响应,因此必须将二者完全分离,否则*α*-鹅膏毒肽会干扰*β*-鹅膏毒肽的测定。

固定甲醇-0.01%甲酸水溶液为流动相,尝试采用多种不同类型的色谱柱进行分离。采用Kinetex F5色谱柱(100 mm×2.1 mm, 1.7 μm)时,*α*-鹅膏毒肽和*β*-鹅膏毒肽完全重叠,而采用颗粒表面带电荷的ACQUITY UPLC CSH C_18_色谱柱(100 mm×2.1mm, 1.7 μm)和Cortecs $C_{18}^{+}$ 色谱柱(100 mm×2.1mm, 1.6 μm)时,二者能够完全分离,分离效果好,但质谱的响应值不高;Kinetex Biphenyl色谱柱(100 mm×2.1mm, 1.7 μm)分离效果次之,二者能达到基本完全分离,并且质谱的响应值较高。因此选择Kinetex Biphenyl色谱柱作为分析用色谱柱。

实验再对不同体积分数的甲酸进行优化,结果见图2。3种鹅膏毒肽在甲醇-水中质谱响应最低,在流动相中加入甲酸,3种鹅膏毒肽的质谱响应值随之增加,当甲酸体积分数为0.0025%~0.005%时质谱响应最高且达到平台,此时*α*-鹅膏毒肽和*β*-鹅膏毒肽基本达到基线分离(分离度*R*_s_为1.4),考虑到较大的缓冲容量易于保持保留时间的稳定,因此选择甲醇-0.005%(v/v)甲酸水溶液作为流动相进行后续试验。标准溶液中3种鹅膏毒肽(0.1 ng/mL)的色谱图见图3。

**图2:**
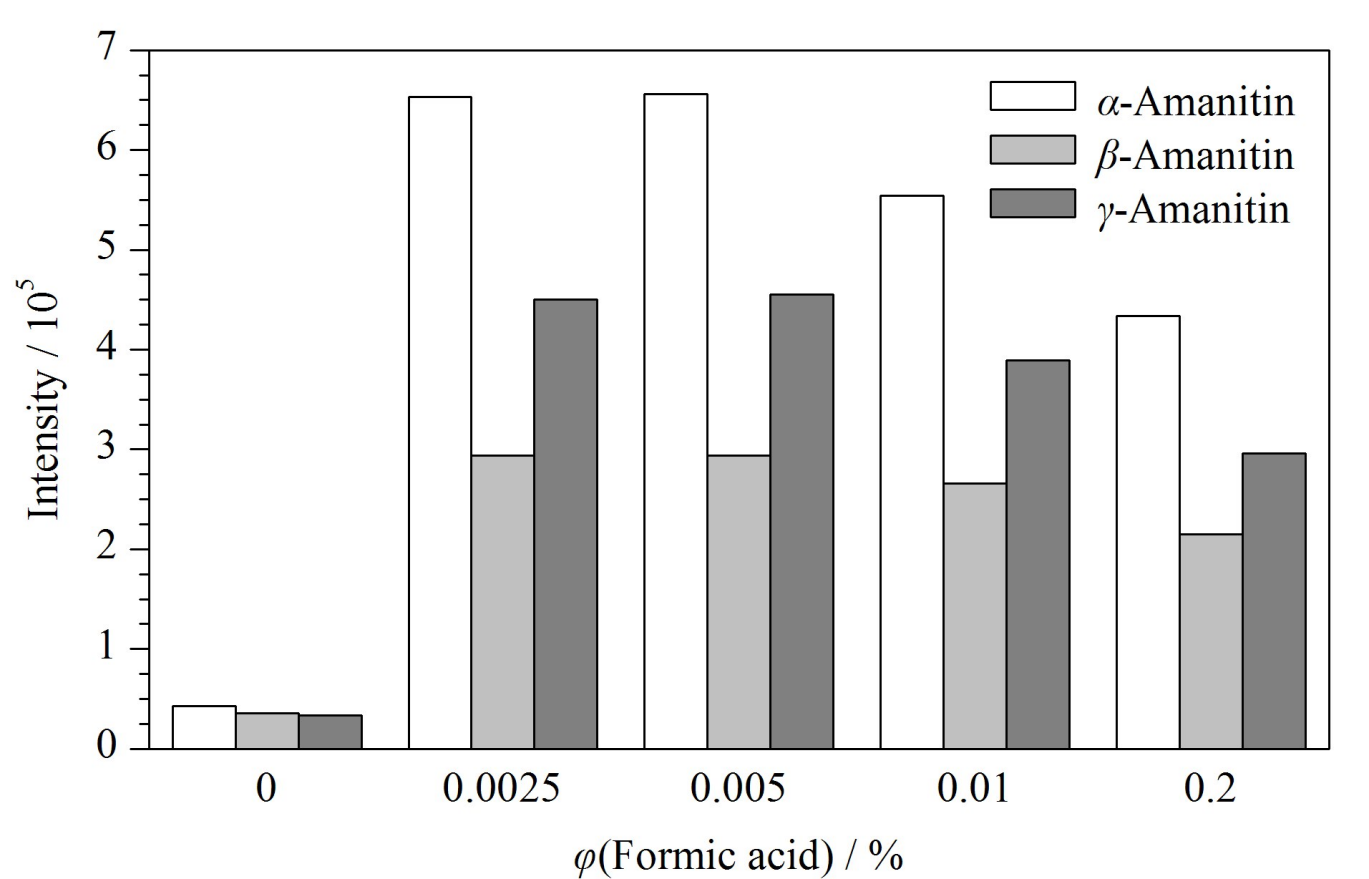
流动相中甲酸的体积分数对3种鹅膏毒肽响应值的影响

**图3:**
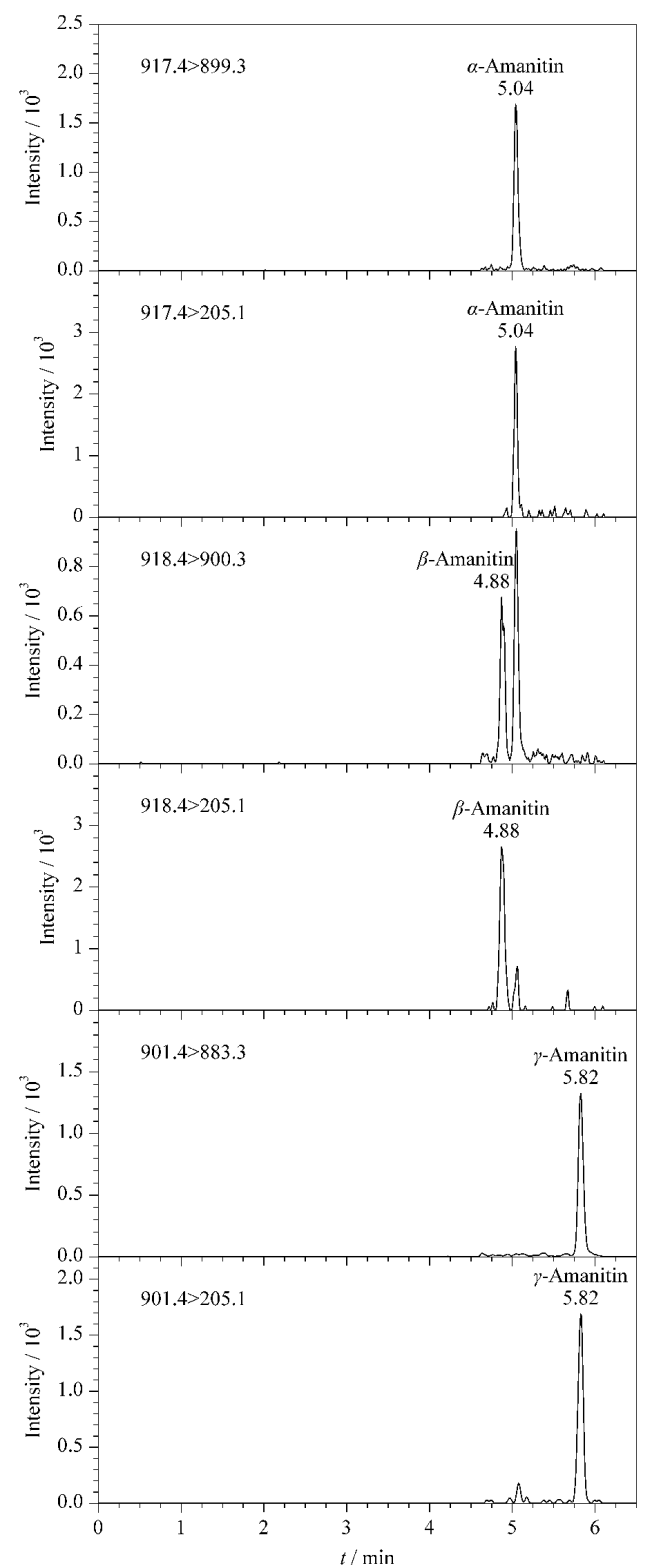
标准溶液中3种鹅膏毒肽(0.1 ng/mL)的色谱图

### 2.3 样品前处理条件的优化

尿液和血浆中鹅膏毒肽的常见样品前处理方法有反相固相萃取法^[13⇓-15,20]^、阳离子交换固相萃取法^[19]^、Turboflow在线固相萃取法^[18,26]^和在线固相萃取法^[22]^,这些方法均属于非特异性净化方法,选择性不强,去除杂质效果不好,而尿液和血浆基质又非常复杂,样品中基质成分会影响检测方法的灵敏度、准确度和精密度。Maurer等^[16]^提出了免疫亲和柱净化方法,选择性强、净化效果好,但需要制备抗原、提取和纯化抗体等烦琐的操作,且*α*-鹅膏毒肽和*β*-鹅膏毒肽的绝对回收率分别仅有61%~63%和57%~58%,方法的灵敏度不高。

本实验采用商品化免疫亲和柱,上样各250 ng的*α*-鹅膏毒肽、*β*-鹅膏毒肽、*γ*-鹅膏毒肽、羧基二羟鬼笔毒肽和二羟鬼笔毒肽,平行测定3次,测得*α*-鹅膏毒肽、*β*-鹅膏毒肽、*γ*-鹅膏毒肽的平均吸附率分别为83.6%、89.6%和86.4%,羧基二羟鬼笔毒肽和二羟鬼笔毒肽的平均吸附率均为0,说明免疫亲和柱特异性较强,只保留3种鹅膏毒肽,而不保留羧基二羟鬼笔毒肽和二羟鬼笔毒肽。取1.0 μg *α*-鹅膏毒肽上样,平行测定4次,测得最大柱容量为670 ng,柱容量较大。

尿液和血浆样品经PBS缓冲溶液(pH 7.3)稀释,确保上样液的pH在6~8范围内,符合免疫亲和柱对上样液pH的要求。样品上样后先用PBS缓冲溶液淋洗除去内源性杂质,然后再用水淋洗除去PBS缓冲溶液中的盐分,避免了磷酸缓冲盐对质谱的不良影响。分别采用甲醇、2%乙酸甲醇溶液、甲醇-丙酮(1∶1, v/v)、乙腈、2%乙酸乙腈溶液和乙腈-丙酮(1∶1, v/v)作为洗脱剂进行3次洗脱,洗脱体积分别为2.0、1.0和1.0 mL,它们对3种鹅膏毒肽的洗脱率见图4。在6种洗脱剂中甲醇-丙酮(1∶1, v/v)的洗脱效果最好,采用3.0 mL洗脱时,3种鹅膏毒肽的绝对回收率分别为99.9%、93.9%、99.3%,明显优于文献^[16]^报道结果。对6份来自不同个体的空白尿液和空白血浆样品进行测定,结果未显示有任何影响3种鹅膏毒肽的干扰峰,净化效果好,方法选择性强。

**图4:**
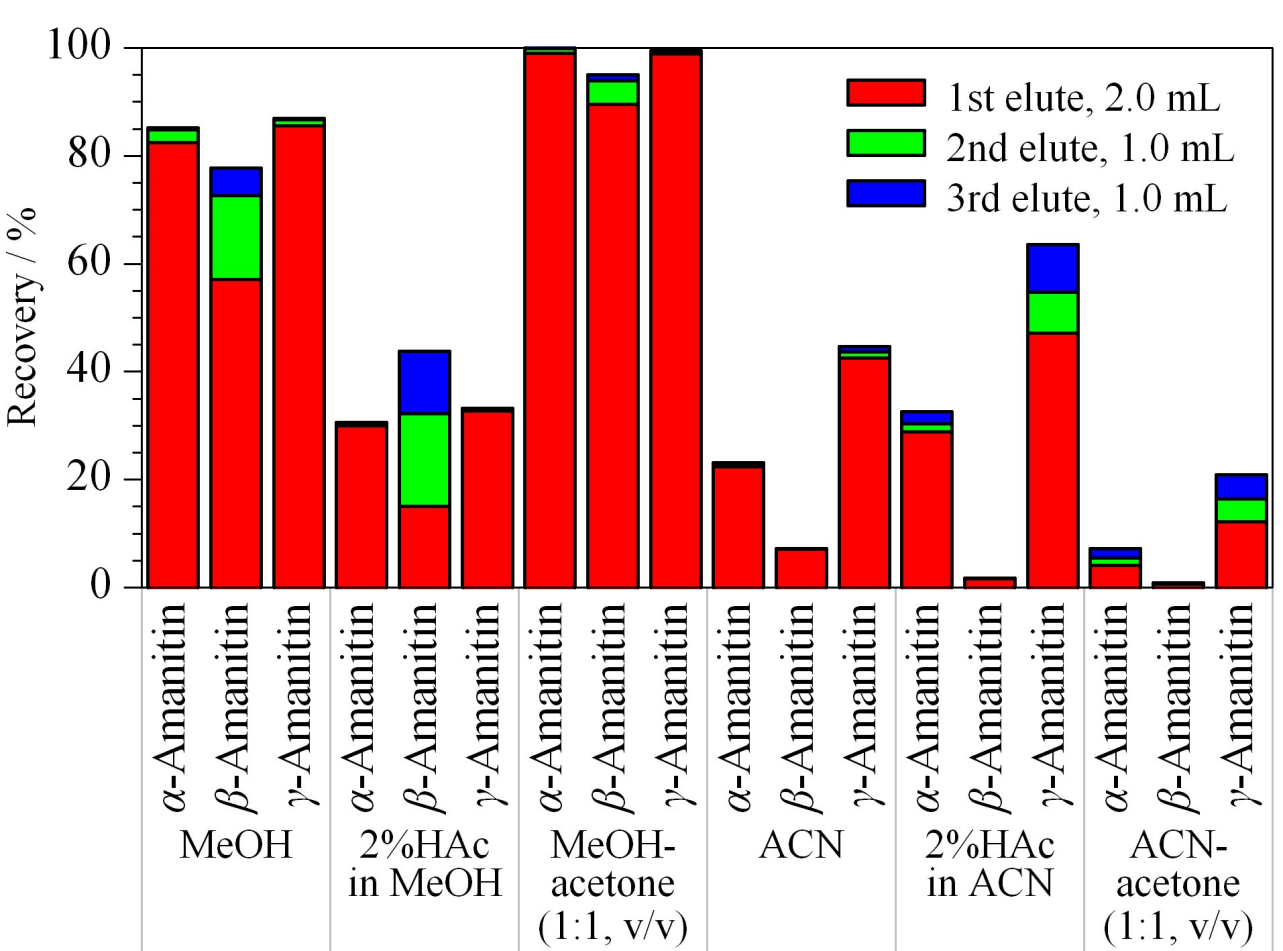
不同类型的洗脱剂对3种鹅膏毒肽洗脱率的影响

### 2.4 标准曲线和线性范围

采用1.4和1.5节条件对1.2节配制的标准溶液进行测定。以定量离子对的峰面积(*Y*)对标准溶液的质量浓度(*X*, μg/L)进行回归(权重取1/*X*), *α*-鹅膏毒肽、*β*-鹅膏毒肽和*γ*-鹅膏毒肽在0.1~200 ng/mL范围内呈良好的线性关系,线性方程分别为*Y*=5.290×10^4^*X*+8.351×10^2^、*Y*=2.332×10^4^*X*+3.228×10^2^和*Y*=5.450×10^4^*X*+6.988×10^2^,相关系数(*r*)均大于0.999。

### 2.5 检出限和定量限

在空白尿液和空白血浆样品中分别加入系列低浓度的待测物进行测定,以分子离子对的信噪比≥3和≥10时的含量分别作为检出限(LOD)和定量限(LOQ),同时定量限还要满足准确度在标称值的±20%之内、精度小于20%的要求。当尿液取样量为2.00 mL时,测得3种鹅膏毒肽检出限和定量限均为0.002 ng/mL和0.005 ng/mL;当血浆取样量为1.00 mL时,测得3种鹅膏毒肽检出限和定量限均为0.004 ng/mL和0.01 ng/mL。方法的灵敏度比文献^[10,13⇓⇓⇓⇓⇓⇓-20]^报道值有数十倍的提高。

### 2.6 基质效应和提取回收率

基质效应和提取回收率采用Matuszewski等^[27]^提出的简易评估方法进行评估,在定量限、低、中、高4种水平下制备了3组样品。第1组是以10%(v/v)甲醇水溶液为溶剂的纯溶剂标准系列,平行样6份;第2组则是取6份来自6个不同个体的空白尿液或空白血浆样品经样品前处理后,加入与第1组相同浓度的待测物制成的尿液或血浆的基质标准系列;第3组采用与第2组相同的空白样品,加入与第1组相同系列浓度的待测成分,样品前处理后得到的尿液或血浆基质加标系列。进样分析分别得到测得每个水平浓度的峰面积(*A*、*B*和*C*),然后分别计算每个水平的基质效应和提取回收率及相应的变异系数,其中:基质效应=*B/A*×100%,提取回收率=*C/B*×100%。

测得尿液和血浆中3种鹅膏毒肽的平均基质效应和提取回收率以及相应的变异系数见附表1(详见 https://www.chrom-China.com)。3种鹅膏毒肽的基质效应和提取回收率分别为92%~108%和90%~103%,变异系数均小于13%,说明来自6个不同个体的尿液均无明显的基质效应,提取回收率又接近100%,因此可以采用溶剂标准外标法定量,方便操作。

### 2.7 准确度和精度

8个连续工作日对定量限、低、中和高等4种水平的尿液和血浆质控样品进行测定,每个水平平行测定2份,计算平均含量、准确度、重复性(RSD_r_)和中间精度(RSD_t_),其中准确度以标称浓度的偏差来表示。以单因素方差解析法(ANOVA)计算重复性和中间精度^[24]^

测得的准确度、重复性和中间精度见附表1,尿液和血浆中3种鹅膏毒肽的准确度分别为-9.4%~8.0%和-13%~8.0%,重复性分别为3.0%~14%和3.9%~9.7%,中间精度分别为3.5%~18%和5.5%~12%,符合欧洲药品管理局(EMEA)关于方法确认的要求^[23]^。

### 2.8 稳定性

系列溶剂标准溶液储存于聚丙烯自动进样瓶中,在(15±5) ℃下至少稳定1个月。加标水平为0.015 ng/mL和8.5 ng/mL的尿液样品和0.030 ng/mL和17.0 ng/mL的血浆样品处理液在(15±5) ℃下在1周内可稳定保存。

在(15±5) ℃下保存,尿液中加标水平为0.015 ng/mL的*α*-鹅膏毒肽和*γ*-鹅膏毒肽在3 d内稳定,*β*-鹅膏毒肽稳定性较差,第1天分解了11%,第3天分解了27%(见图5a);尿液中加标水平为8.5 ng/mL的*γ*-鹅膏毒肽在15 d内稳定,*α*-鹅膏毒肽和*β*-鹅膏毒肽则只在3 d内稳定(见图5b)。在4 ℃下保存,尿液中*α*-鹅膏毒肽和*γ*-鹅膏毒肽至少在15 d内稳定,而低水平*β*-鹅膏毒肽在3 d内稳定,高水平*β*-鹅膏毒肽在6 d内稳定。尿液中3种鹅膏毒肽于-20 ℃保存1个月后再测定,没有明显分解;2个月后再测定,*α*-鹅膏毒肽和*γ*-鹅膏毒肽稳定,而*β*-鹅膏毒肽则有分解。

**图5:**
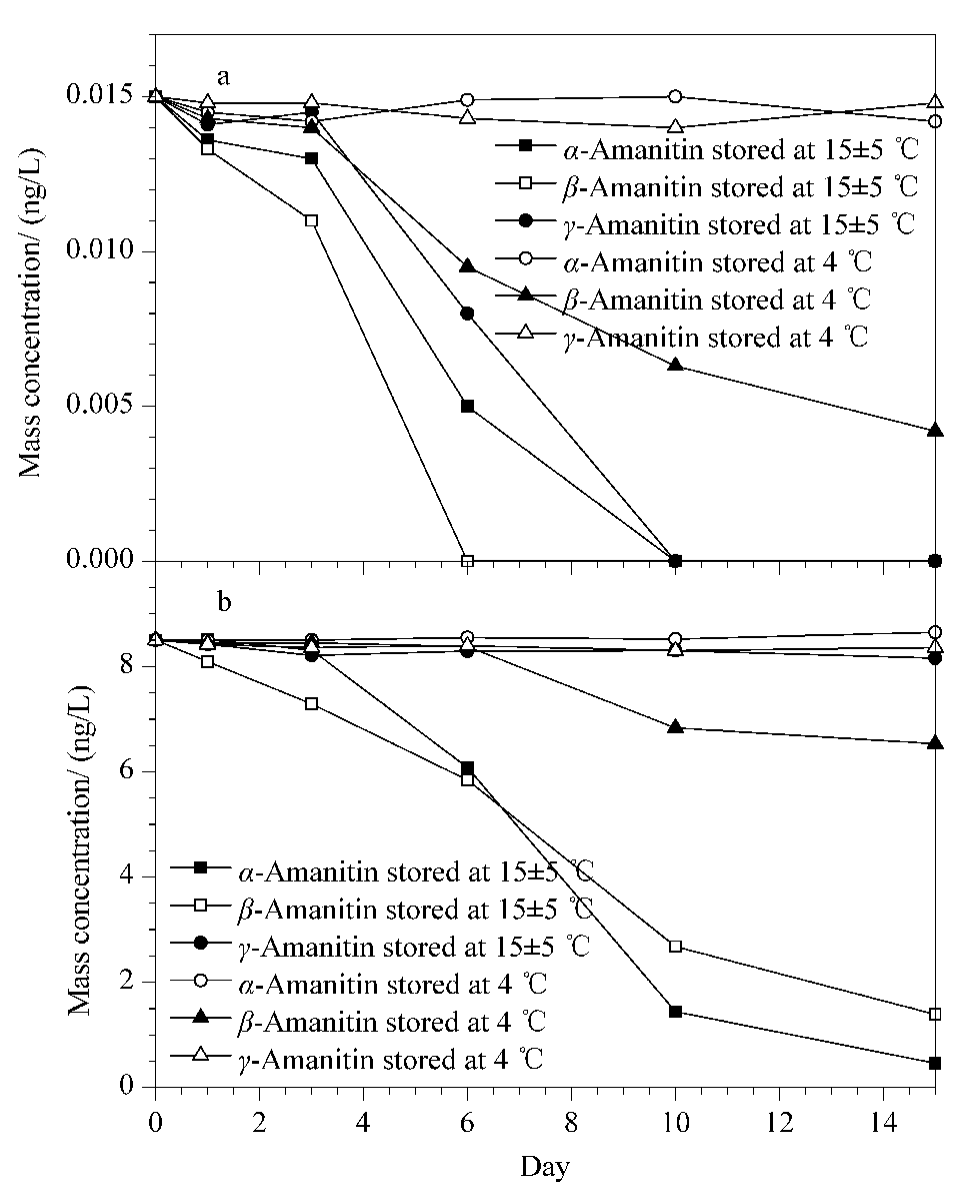
空白加标尿液在(15±5) ℃和4 ℃条件下的稳定性试验

血浆样品中3种鹅膏毒肽比较稳定,在(15±5) ℃、4 ℃和-20 ℃下保存3个月没有明显的分解,与文献^[14]^报道相一致。甚至血浆样品在-20 ℃下冷冻保存7个月,*α*-鹅膏毒肽无明显分解。

经过3个过夜冷冻(-20 ℃, 4 h)室温融化过程后,加标水平为0.015 ng/mL、8.5 ng/mL的尿液样品和0.030 ng/mL、17.0 ng/mL的血浆中3种鹅膏毒肽没有明显分解。

### 2.9 实际应用

2020年6月5日浙江省温州市发生一起食用野生蘑菇的中毒事件,1人中毒,患者于6月5日中餐和晚餐食用了野生蘑菇,6日就医时出现肝损害症状。6月11日中午采集尿液(晚餐摄入野生蘑菇后138 h),按本法进行前处理,测得尿中*α*-鹅膏毒肽的含量为0.0067 ng/mL, *β*-鹅膏毒肽的含量为0.0059 ng/mL, *γ*-鹅膏毒肽未检出。尿液的MRM色谱图见图6a。

**图6:**
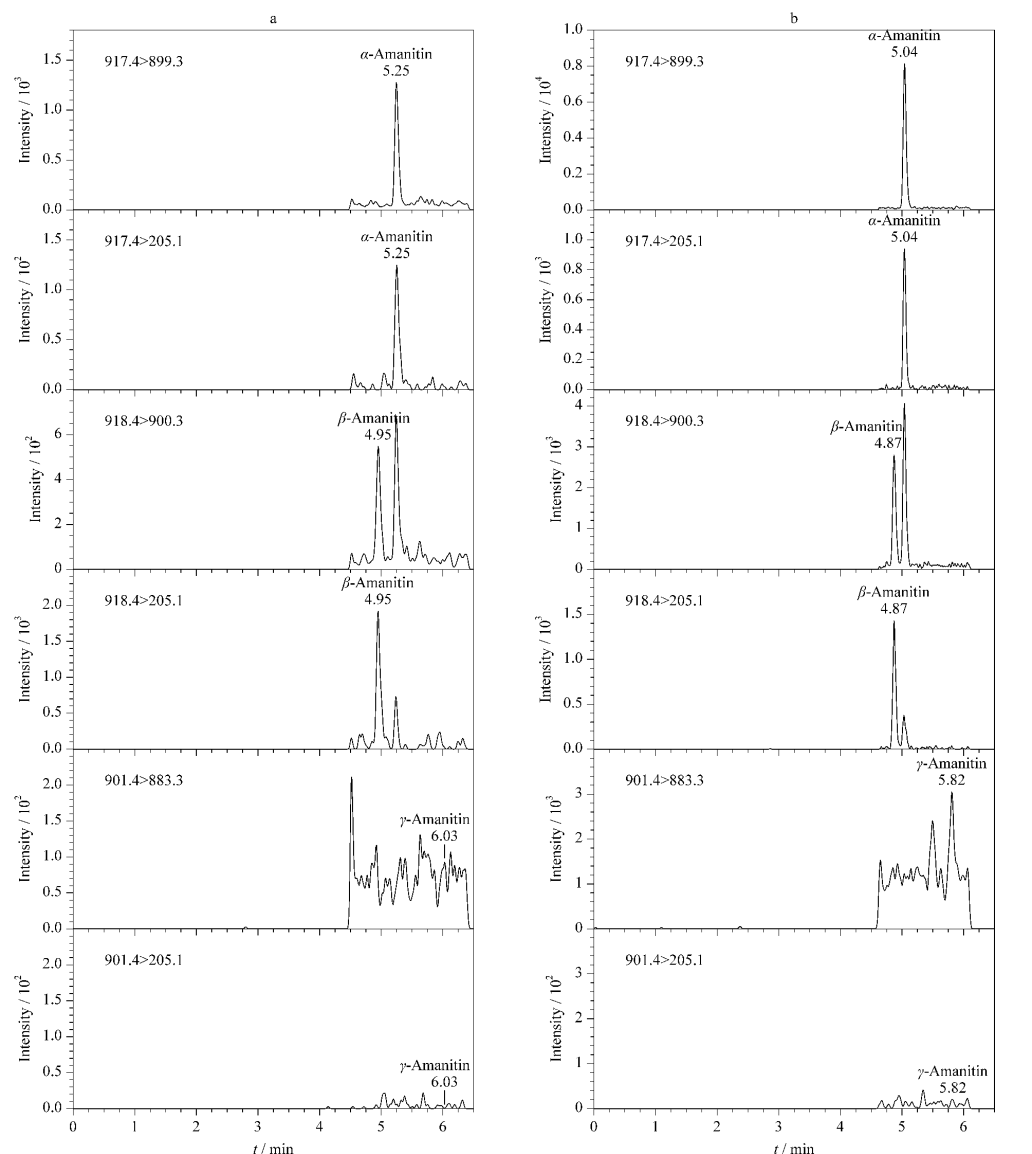
实际中毒(a)尿液样品和(b)血浆样品的MRM色谱图

2021年3月对-20 ℃冷冻保存7个月的血浆样品(采样时间距离摄入野生蘑菇20 h,浙江省疾病预防控制中心友情赠予)进行测定,样品取200 μL,按本文方法处理后定容至100 μL,测得*α*-鹅膏毒肽的含量为0.225 ng/mL,与浙江省疾病预防控制中心原测定值0.20 ng/mL一致,同时还测得*β*-鹅膏毒肽的含量为0.203 ng/mL, *γ*-鹅膏毒肽未检出,其MRM色谱图见图6b。

## 3 结论

本文建立了高灵敏测定尿液和血浆中3种鹅膏毒肽的超高效液相色谱-三重四极杆质谱分析方法。通过对色谱分离条件、质谱检测条件和样品前处理方法的优化,方法得到了较高的检测灵敏度;通过对免疫亲和柱样品净化效果和基质效应的评估,证明了样品前处理方法的有效性。方法学验证结果表明,本法灵敏度高、选择性好、定量准确、精密,已应用于鹅膏毒肽实际中毒样品的检测,得到满意结果。本法已成功解决中毒患者尿液和血浆中超痕量鹅膏毒肽的检测难题,对于疑似中毒病人的早诊断、早治疗、降低死亡率都具有非常重要的现实意义,也为今后开展此类毒素的毒理作用及机体代谢规律的研究提供了可靠的技术支撑。
